# A 3D journey on virtual surfaces and inner structure of *ossa genitalia* in Primates by means of a non-invasive imaging tool

**DOI:** 10.1371/journal.pone.0228131

**Published:** 2020-01-30

**Authors:** Federica Spani, Maria Pia Morigi, Matteo Bettuzzi, Massimiliano Scalici, Monica Carosi

**Affiliations:** 1 Department of Sciences, Roma Tre University, Rome, Italy; 2 Department of Physics and Astronomy, University of Bologna, Bologna, Italy; 3 Historical Museum of Physics and "Enrico Fermi" Study and Research Center, Rome, Italy; 4 National Institute of Nuclear Physics, Rome, Italy; Liverpool John Moores University, UNITED KINGDOM

## Abstract

Novel bio-imaging techniques such as micro-Computed Tomography provide an opportunity to investigate animal anatomy and morphology by overcoming limitations imposed by traditional anatomical drawings. The primate genital bones are complex anatomical structures whose occurrence in both male penis (*baculum*) and female clitoris (*baubellum*) may be difficult to assess in individual cadavers. We tested a 3-step methodological protocol, including different techniques ranging from inexpensive/simple to more expensive/sophisticated ones, by applying it to a sample of primate species, and resulting in different levels of data complexity: (1) presence/absence manual palpation method; (2) 2D X-ray plates; 3) 3D micro-CT scans. Manual palpation failed on 2 out of 23 specimens by detecting 1 false negative and 1 false positive; radiography failed once confirming the false positive, however firmly disproved by micro-CT; micro-CT analysis reported the presence of 9 *bacula* out of 11 male specimens and 1 *baubellum* out of 12 female specimens. A different *baculum* position was identified between strepsirrhine and haplorrhine species. We also aim to assess micro-CT as a non-invasive technique providing updated anatomical descriptions of primate *ossa genitalia*. Micro-CT 3D volumes showed the surface of some bones as rough, with a jagged appearance, whereas in others the surface appeared very smooth and coherent. In addition, four main types of bone internal structure were identified: 1) totally hollow; 2) hollow epiphyses and solid diaphysis with few or several channels inside; 3) totally solid with intricate Haversian channels; 4) totally solid with some channels (structure of single *baubellum* scanned). *Ossa genitalia* appeared as a living tissue having its own Haversian-like channels. The high resolution of micro-CT 3D-images of primate genital bones disclosed additional form variability to that available from genital bone 2D images of previous studies, and showed for the first time new internal and external morphological characters. Moreover, micro-CT non-invasive approach proved appropriate to recover much of scientific knowledge still hidden and often neglected in both museum specimens and primate cadavers only destined to necropsy.

## Introduction

Novel radiological techniques have greatly advanced our knowledge in animal morphology, allowing to quantify internal anatomy with higher degree of accuracy, also using non-invasive approaches. In the past, anatomical studies were based on bi-dimensional drawings and/or photographs that allow to obtain limited information about size and shape. To date, bio-imaging techniques overcome these methodological limits since they produce virtual 3D models which, in addition, provide the access to more detailed descriptors of morphological variation. The availability of micro-CT imaging has increased over the last decade and has shown its utility in many biological [1–3] and medical applications [4–6]. In comparison with other imaging techniques, the strengths of micro-CT lie in its high-resolution imaging, scanning efficiency, relative low cost, and totally non-destructive approach reaching a whole 3D inspection of the sample [7]. Micro-CT provides a reliable working platform enabling numerous morphological and functional imaging applications, suitable for small animals or other biological structures. This is the case of the primate small genital bones, known as *baculum* in males and *baubellum* in females.

Morphological evolution of *ossa genitalia* in mammals represents a still unresolved old puzzling enigma [8,9] that is attracting renewed interest among scientists [10–15]. Genital bones are considered as heterotopic bones [16–21], that is they do not belong to the skeleton itself, rather they are accessory structures (such as the *os cordis* in deer and bovids heart, or the *os palpebre* in the Crocodilian eyelids).

*Baculum* (Latin for “scepter”), also known as *os penis*, *os priapi*, or penile bone, usually lies inside the distal half of the penis and it has been described in 7 mammalian orders: Eulipotyphla (including shrews, moles and solenodons), Carnivora (cat-like and dog-like), Chiroptera (Yangochiroptera and Yinpterochiroptera), Primates (great and lesser apes, monkeys and prosimians), Rodentia (mice, rats and porcupines), Lagomorpha (hares and rabbits), and Afrosoricidae (tenrecs and golden moles) [20]. The female counterpart of the penile bone is the *baubellum* (Latin for “gem, jewel”), also known as the *os clitoris* or clitoral bone, that usually lies inside the distal tip of female clitoris, and it has been found in 4 mammalian orders so far: Chiroptera, Carnivora, Rodentia and Primates [22].

Among primates, one of the first mentions of a *baculum* was by the French naturalist and explorer Alfred Grandidier [23]. In his scientific treatise on mammals of Madagascar, several anatomical boards of external genitals, dissected from some indrid species (Indridae), are found, as well as sketches of the *ossa genitalia*. He wrote about *Avahi laniger* (Gmelin, 1788) penile bone: “[…] *Le gland est soutenu par un osselet étendu au-dessus de l'urèthre et terminé en avant par une extrémité fourchue*” (pag. 273) (“[…] The glans is supported by a bone extending on the urethra and ending in the distal half as a fork”). In the same treatise the first mention of *baubellum* is also found (*Propithecus edwarsi* A. Grandidier, 1871): “*Chez les Propithèques*, *l'ouverture vulvaire est en grande partie recouverte et cachée par le clitoris qui […] un très-petit osselet soutient l'extrémité de cet organe*” (pag. 275) (“In *Propithecus*, the vulva opening is largely covered and hidden by the clitoris, […] a small bone supports the extremity of this organ”). Afterwards, several authors have been attracted by the study of the overall occurrence and anatomy of genital bones in Eutheria [24–26], more so in Primates [27–45]. Nevertheless, photographic evidences are extremely rare, and these precious, fascinating, mostly old scientific reports, all suffer from the use of drawing as, in most cases, the only available tool to get and store two-dimensional information.

From an evolutionary perspective, merging the apparent multitude of *bacula* and *baubella* forms (*i*.*e*., size+shape) into a univocal functional interpretation, may potentially suffer from an oversimplification [46]. In fact, a positive and significant relationship between *baculum* length and prolonged intromission patterns during copulation [43,45,47,48], represents the only available data so far, supporting just one of the few existing hypotheses about *baculum* adaptive function [22,24,44,49]. Although informative, this analysis is nevertheless firstly limited to *bacula* only, and secondly restricted to just one morphometric one-dimensional parameter such as length, with all potential information contained in bone shape variability neglected.

In this study we firstly aim to identify the feasibility and potential of a 3-step methodological protocol to reliably detect *ossa genitalia* in primates by means of non-invasive techniques. We compared data acquisition methods characterized by a progressive increase in both accuracy/completeness and costs. Our protocol goes from the obtainment of simple occurrence data, to virtual volume and inner structure data by applying: (1) manual palpation, (2) 2D projectional radiography (x-ray plates), and (3) 3D micro-CT. Secondly, we show how 3D high resolution virtual reconstructions of penile and clitoral bones reveal to be a precious source of knowledge in primates as well, disclosing for example (i) exact spatial position of bones inside tissues of external genitals, (ii) external and inner morphological variability, and (iii) the importance of relying on detailed 3D data in order to make meaningful comparative studies among primate species (as well as other mammal orders). 3D models of *ossa genitalia* have been stored in a permanent archive (morphosource.org), available for further functional analyses.

## Materials and methods

### Samples

Samples of external genitals came from several sources. From the historical (XIX-XX century) primate wet collection of the Natural History Museum of “La Specola” in Florence, Italy (NHMLS) [50] we obtained 13 external genitals (separated *in loco* from the whole body by cutting at the base of penis and clitoris close to the pubic bone surface) and one *baculum* (as an already extracted bone), all accounting for 13 different primate species. In addition, 8 fresh samples of external genitals belonging to 5 different species were obtained from corpses at Istituto Zooprofilattico Sperimentale di Lazio e Toscana (IZSLT, Rome, Italy). All Italian Istituti Zooprofilattici Sperimentali (II. ZZ. SS.) usually receive primate corpses (dead for natural causes or trauma/disease) from zoos and/or research institutes, in order to carry out necroscopic investigations aimed at identifying possible risks of zoonoses. Consequently, we established a collaboration with the IZSLT in order to be notified when a primate body underwent necropsy, and therefore the cut off external genitals were available. Generally, after necropsy animal remains are disposed of and most of the anatomical information related to the specimens is completely lost. One additional sample came from the Civic Museum of Zoology (CMZ) in Rome (Italy). In this case, the specimen was a female hybrid orangutan (*Pongo pygmaeus x abelii*) near to be taxidermied. We were able to obtain external genitals thanks to the taxidermist before the process began. Overall, we collected 23 samples of external genitals belonging to 19 different primate species, 5 of which belonging to the suborder strepsirrhines (N = 5 males) and 18 belonging to the suborder haplorrhines (N = 12 females, and N = 6 males) (S1 Table). As for museum specimens, neither skins nor skeletons but only wet samples could be considered as a source of genital bones, in that skins and skeletons could both be source of falsified data, specifically of false absences. In fact, skins, by definition, no longer include either soft tissues or bones inside them, while skeletons may have lost such small bones after the cleaning procedure (often boiling bones to detach tissues).

### 3-step methodological protocol

First step: the manual palpation method. Both penises and clitorises were exposed to a double palpation by two different operators (FS and MC) who touched the genitals with thumb and forefinger of the same hand (a) along medio-lateral axis and (b) along anteroposterior axis. Palpation type (b) may overcome problems related to the palpation type (a) during which a tendon or a ligament may be construed as a small bone instead. Second step: 2D projectional radiography (X-ray plates shot with Arcom Simply system for veterinary radiodiagnostics; 20 mA—40 kV setting, at “Enrico Fermi” veterinary clinic, Rome, Italy). This step was used to either verify data coming from the manual palpation method or select specimens that, possibly confirmed as containing a bone, were suitable for the step 3 tomography protocol. Third step: cone-beam micro-CT, assembled at the Department of Physics and Astronomy of Bologna University (Italy; http://www.fisica-astronomia.unibo.it/it/dipartimento/servizi-e-strutture/strutture/laboratori-di-ricerca-1/indagini-con-i-raggi-x/radiografia-digitale-e-tomografia-3d). The computed tomography was used to scan samples and obtain 3D virtual volumes of genital bones. For all three steps, no bone extraction from tissues was necessary and integer genitals were also used to investigate both their external and internal morphology.

The total time needed to detect and visualize genital bones depends on the size of each bone. On average, the time required to carry out the 3-step methodological protocol is 3 hours and 50 minutes per bone (see Table 1).

**Table 1:** Duration of 3-step methodological protocol.

3-step methodological protocol		
Step n°		Time (mins)
1		5
2		10
3		215
3.1	alignment^a^	60
3.2	scan^b^	75
3.3	reconstruction	60
3.4	3D rendering	10
**Total time**		230

Time needed for each step (and total time) of the suggested protocol for detecting and visualizing *ossa genitalia*. ^a^ = time needed for the correct alignment is far below in commercial lab-based micro-computed tomography systems. ^b^ = the scan times are halved in commercial lab-based micro-computed tomography systems (with the same scanning parameters used).

### Micro-CT setup and procedure

**Fig 1:**
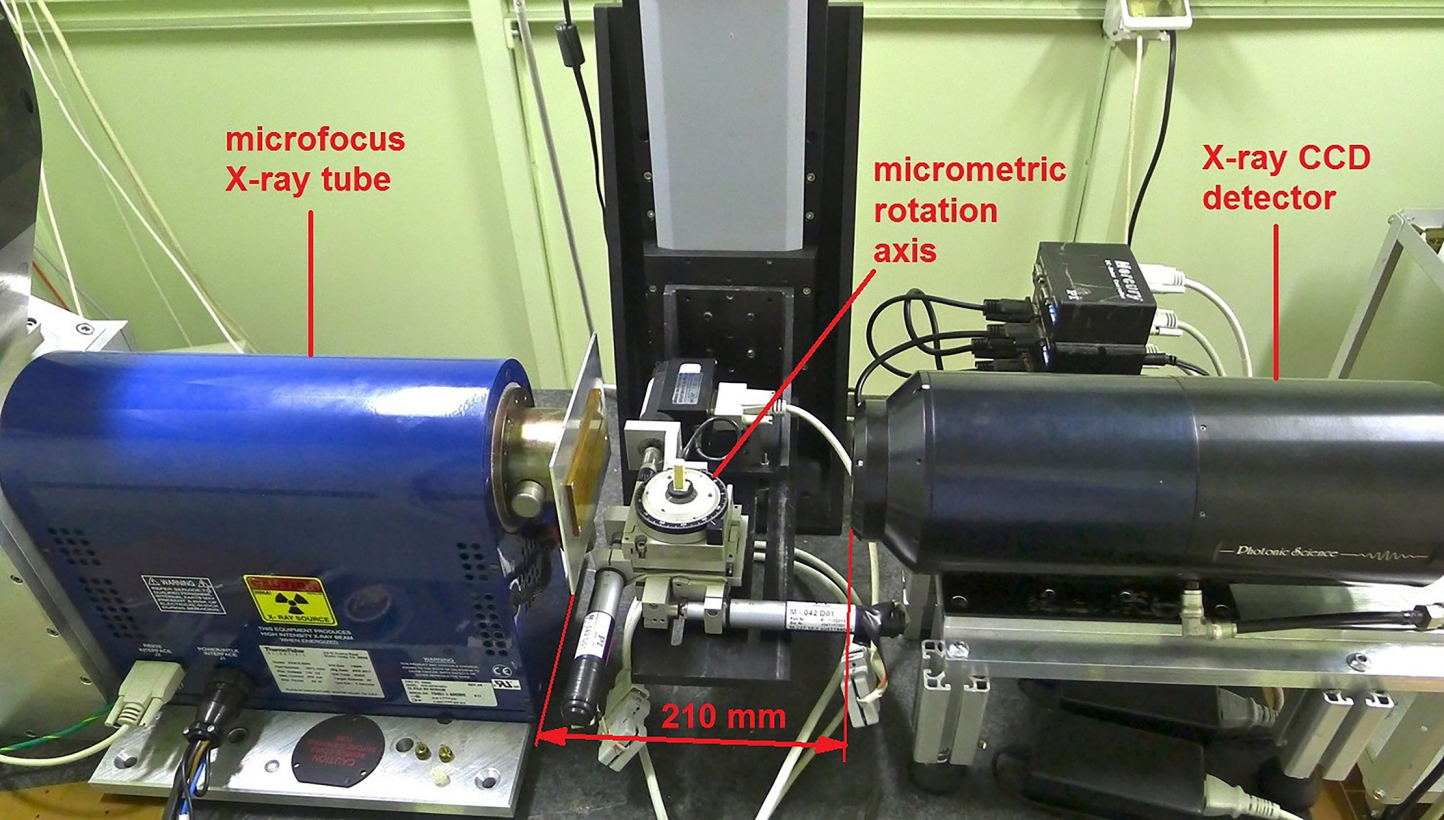
Micro-Computed tomography system. The cone-beam micro-CT system, assembled at the Department of Physics and Astronomy of Bologna University (Italy), and used to scan and obtain 3D virtual volumes of genital bone samples.

The set-up of the micro-CT scanner (Fig 1) is based on a microfocus X-ray tube (Thermo Scientific/Kevex PXS10-65W, Scotts Valley, CA, USA; operating voltage range: 45–130 kV; maximum beam current: 0.5 mA; minimum focal spot: 5 μm) and a Photonics Science (Saint Leonards-on-sea, United Kingdom) VHR1:1 Charge Coupled Device (CCD) camera (4008 × 2672 pixels, 9 μm pixel size) with a 36 × 24 mm^2^ field of view (FOV). This camera features a high-resolution phosphor screen directly coupled to the CCD by means of a fibre-optic plate that protects the sensor against radiation damage. The sample manipulator consists of a high-precision rotary table (model M-037 by Physik Instrumente, Karlsruhe, Germany), on which the sample is positioned, and a tip-tilt platform (model M-042 by Physik Instrumente, Karlsruhe, Germany) to assure the correct alignment between the vertical rotation axis and the pixel matrix of the detector. The sample manipulator is mounted on a vertical translation axis (model M-415 Physik Instrumente, Karlsruhe, Germany) that makes it possible to analyse, in successive steps, objects with a vertical size larger than the CCD camera’s FOV [51]. Table 2 shows the range of setting parameters used to scan the genital samples.

**Table 2:** Scanning parameters.

Parameter	Setting
Voltage (kV)	60–100
Beam current (μA)	80–200
Al filter (mm)	none/1
N° projections	900
Total rotation angle	360°
Exposure time (s)	1
Voxel size (μm)	10–14

Setting parameters used to scan samples of primate genital bones with micro-CT.

The step-by-step scanning procedure consists of: (a) preliminary check of correct alignment of the system; (b) X-ray acquisition in order to verify presence of bone as identified for its high radiopacity, and to find the optimal scanning parameters; (c) trials of rotation to set sample position within the capture field; (d) setting correct parameters for each sample and launch the scanning procedure. A single scan lasts about 75 minutes at the end of which, samples are returned to their original ethanol containers.

Final phases are the post-processing and the reconstruction phase that were performed with the proprietary software PARREC (it uses parallel processing on several cores and CPUs). Radiographic projections are normalized with respect to the white field and dark field (respectively the image acquired with no object and the image with X-rays off) producing the so called atenrads whose values are natural logarithm of the intensity transmission values ranging from 0 to 1 and corresponding to the different attenuation of the object along the X-ray path. After the adjustment of the optimal rotation centre on the images the actual reconstruction process starts and atenrads are first properly filtered in the frequency domain and then back projected with the cone beam algorithm. The result is a set of sections (in this case vertical sections) called *slices* whose values are proportional to density and atomic number of the materials (in this case bone and soft tissue).

**Fig 2:**
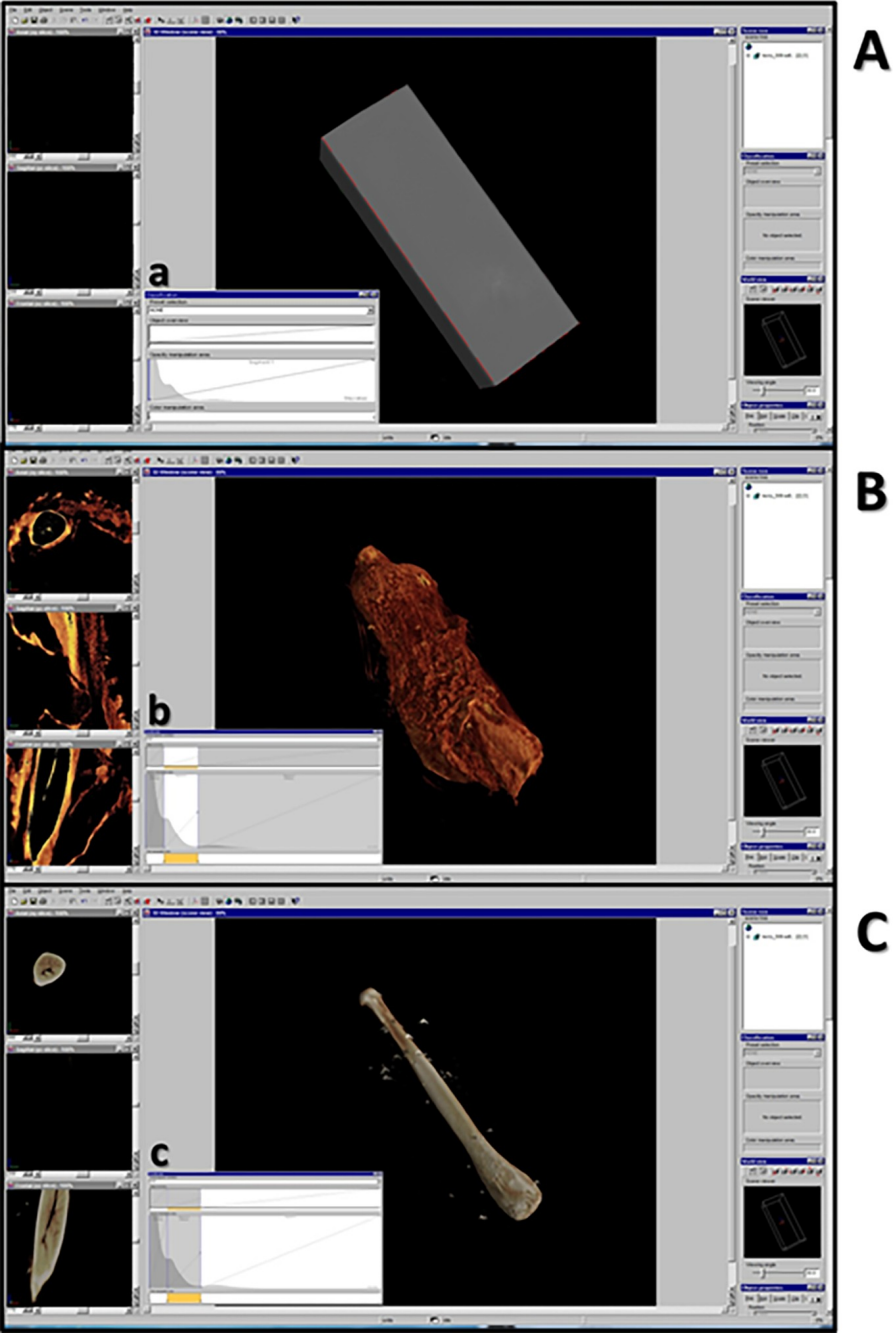
Rendering and data processing commercial software: VGStudio Max 1.0. The user interface showing the reconstructed 3D volume of genital bone in *Loris lydekkerianus*. A: the reconstructed 3D volume appears as a gray and opaque parallelepiped. The gray value histogram (a) with air, soft tissue and bone peaks is shown. B: selecting the segment of the gray value histogram (b) corresponding to air peak, and disabling it, makes soft tissues visible; on the left, there are 2D views of three orthogonal cross sections: axial (up, for x-y slices); sagittal (middle, for y-z slices); frontal (down, for x-z slices). By scrolling bars under each orthogonal view, it is possible to choose the single slice to be visualized. C: selecting and disabling segment of the gray value histogram (c), corresponding to soft tissue peak, make the bone visible.

The stack of slices is loaded with VGStudioMax 2.1, a rendering and data processing commercial software capable to show a 3D data representation. A “histogram”-based segmentation (Fig 2) of different materials (in our case bone and soft tissue) visualizes a virtual three-dimensional scene and allows the grey-level adjustment. In particular, by using the classification tool it is possible to enable or disable different segments of the grey-level histogram corresponding to tissues of different density in the scanned sample, thus bringing out the genitals and/or the bone inside.

**Fig 3:**
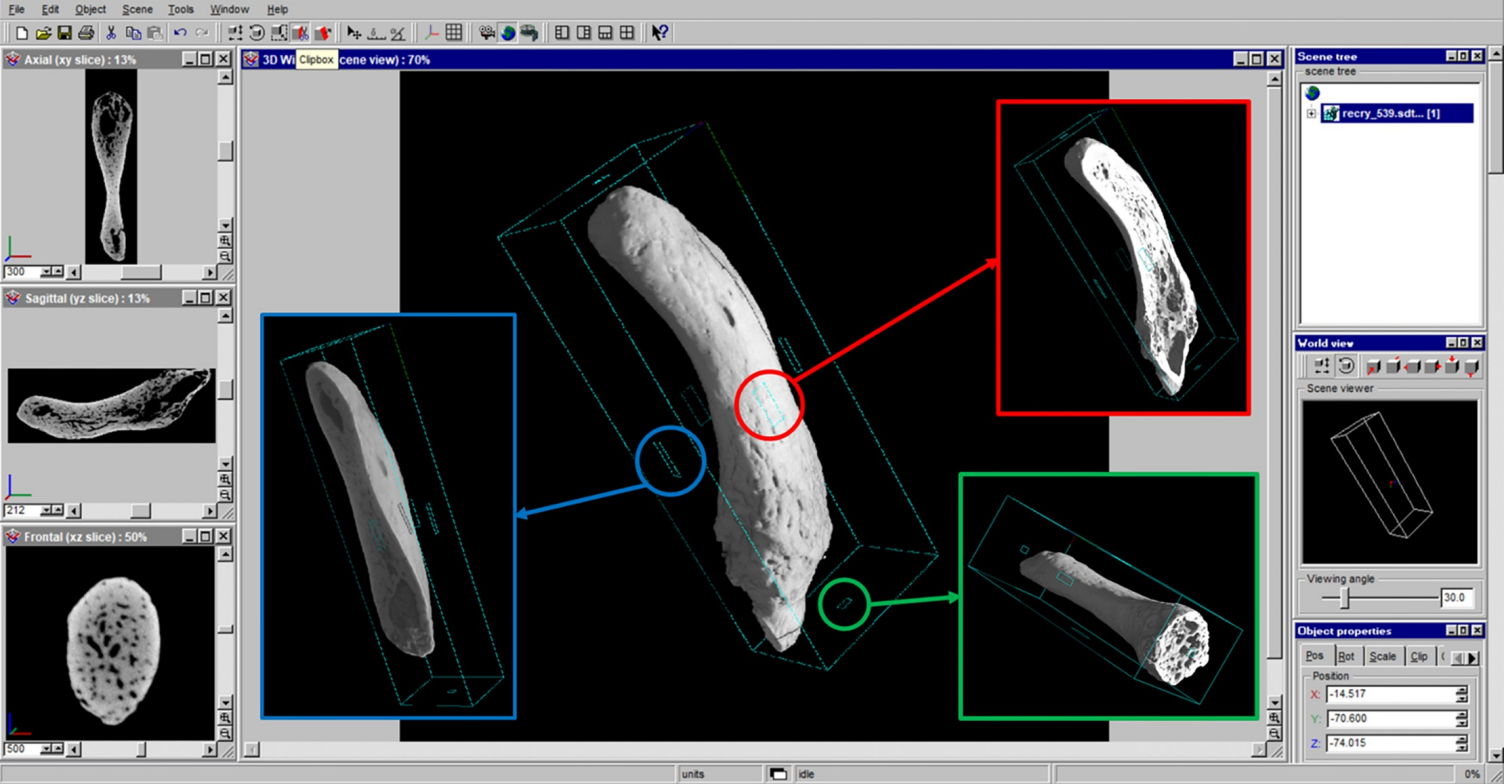
VGStudio Max 1.0 clipbox mode. The user interface showing the cyan bounding box of clipbox mode. Squared handles (placed on each side of the bounding box) are active areas and moving the cursor into them the object may be clipped. 3D orthogonal cross sections obtained are axial (blue box), sagittal (red box), frontal (green box). On the left, there are 2D views of the same orthogonal cross sections: axial (up), sagittal (middle) and frontal (down).

In addition, the rendering software allows the manipulation (translation, rotation and virtual slicing) of the object volume. It was therefore possible to virtually dissect scanned bones, in order to investigate their internal structures. In fact, in the “clipbox mode” the object may be clipped along the desired clipping direction, thus obtaining 3D orthogonal cross sections: axial (Fig 3, blue box), sagittal (Fig 3, red box), and frontal (Fig 3, green box). On the left of the user interface 2D-image viewing windows also appear showing the three orthogonal cross sections of the sample.

Despite linear length data do not require the application of 3D techniques to be sampled, 3D reconstructions allow to collect linear data for museum specimens that cannot undergo a destructive sampling. By using the distance measurement tool, start and end points were set on the distal and proximal tips, respectively. Lengths were blindly sampled for three times by the same operator (FS) and means were calculated.

Limited to two fresh samples (*baculum* of *Mandrillus sphinx*, Linnaeus, 1758, and *baubellum* of *Sapajus apella*, Linnaeus, 1758; provider IZSLT, non-museum samples), the bone was extracted in order to directly investigate both orientation and position inside tissues. We followed a five-step extraction protocol: 1) a transversal cut of penis at the proximal end of bone; 2) a longitudinal cut, from the urethra external opening (distal end of genitals) until the proximal end of bone, therefore opening the whole penis in two halves; 3) several cuts in order to separate the bone from the penis; 4) boiling the extracted bone in water for 5 minutes; 5) detaching all tissues from bone to obtain a clean and smooth surface.

## Results

Validation of our 3-step protocol is shown in Table 3. If we consider micro-CT (step 3) as the most sophisticated and sensitive technique, manual palpation failed on 2 out of 23 specimens, with a false negative and a false positive, while radiography (step 2) failed once. The latter sample, interestingly, though corresponding to the false positive by manual palpation, was firmly proved wrong by the true absence detected by the micro-CT (see discussion).

**Table 3:** Validation of 3-step protocol.

Species	Suborder	Sex	Palpation	X-rays	micro-CT
***Galagoides demidoff***	**S**	**M**	**0**	**1**	**1**
(G. Fischer, 1806)					
***Galago gallarum***	**S**	**M**	**1**	**1**	**1**
Thomas, 1901					
***Loris lydekkerianus***	**S**	**M**	**1**	**1**	**1**
Cabrera, 1908					
***Loris tardigradus***	**S**	**M**	**1**	**1**	**1**
(Linnaeus, 1758)					
***Otolemur crassicaudatus***	**S**	**M**	**1**	**1**	**1**
(É. Geoffroy, 1812)					
*Alouatta* sp.	H	F	0	0	0
Lacépède, 1799					
*Ateles paniscus*	H	F	0	0	0
(Linnaeus, 1758)					
*Callithrix penicillata*	H	M	0	0	0
(É. Geoffroy, 1812)					
*Cercocebus atys*	H	F	0	0	0
(Audebert, 1797)					
***Chlorocebus aethiops***	**H**	**M**	**1**	**1**	**1**
(Linnaeus, 1758)					
*Macaca fascicularis*	H	F	0	0	0
(Raffles, 1821)					
*Macaca fascicularis*	H	F	0	0	0
(Raffles, 1821)					
*Macaca fuscata*	H	F	0	0	0
(Blyth, 1875)					
*Macaca fuscata*	H	F	0	0	0
(Blyth, 1875)					
*Mandrillus sphinx*	H	F	0	0	0
(Linnaeus, 1758)					
***Mandrillus sphinx***	**H**	**M**	**1**	**1**	**1**
(Linnaeus, 1758)					
*Pan troglodytes*	H	M	0	0	0
(Blumenbach, 1799)					
***Papio cynocephalus***	**H**	**M**	**1**	**1**	**1**
(Linnaeus, 1766)					
*Pongo pygmaeus x abelii*	H	F	0	0	0
***Saguinus niger***	**H**	**F**	**1**	**1**	0
(É. Geoffroy, 1803)					
***Saguinus niger***	**H**	**M**	**1**	**1**	**1**
(É. Geoffroy, 1803)					
***Sapajus apella***	**H**	**F**	**1**	**1**	**1**
Linnaeus, 1758					
*Tarsius bancanus*	H	F	0	0	0
Horsfield, 1821					

Presence (1) and absence (0) of genital bones in each specimen resulting from double palpation method (along latero-lateral and anteroposterior axis; Palpation), X-ray plates (X-rays), and micro-CT scans (micro-CT). In bold, selected specimens for micro-CT scans. Species are listed following a phylogenetic order (Suborder): firstly strepsirrhines (S) and after haplorrhines (H) and for every species sex (F = female; M = male) is also reported.

**Fig 4:**
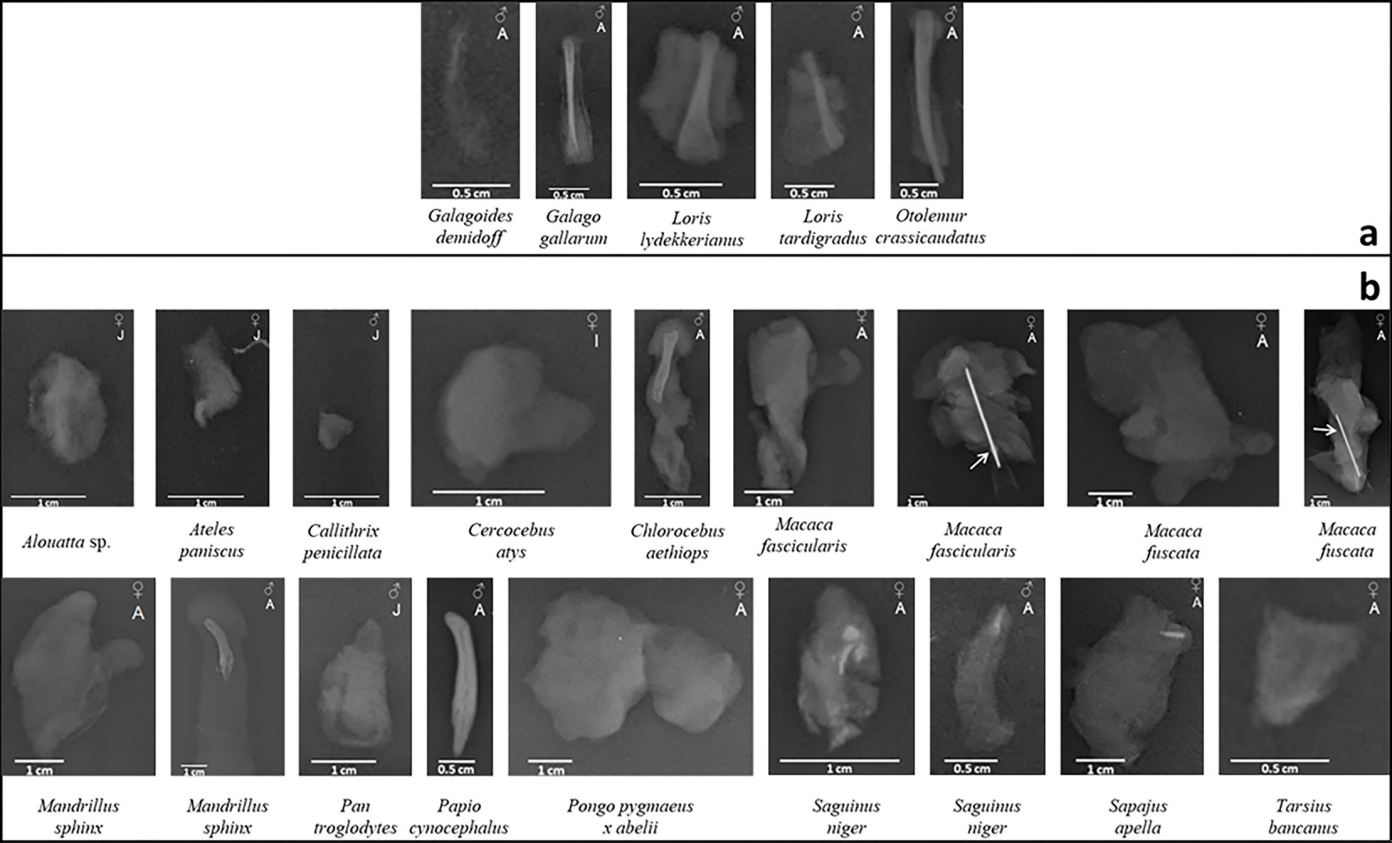
X-ray plates. X-ray plates of 23 specimens of primate external genitals, among which one genital bone *(Papio cynocephalus)*. a = strepsirrhine species; b = haplorrhine species. Only for specimen *Macaca fascicularis* and *Macaca fuscata* a needle (indicated by the arrow) was used to stabilize them.

Micro-CT validated X-rays showing the presence of a *baculum* in 9 out of 11 male specimens, *i*.*e*., 5 strepsirrhines and 4 haplorrhines (as shown in Fig 4):1) *Galagoides demidoff*; 2) *Galago gallarum*; 3) *Loris lydekkerianus*; 4) *Loris tardigradus*; 5) *Otolemur crassicaudatus*; 6) *Chlorocebus aethiops*; 7) *Mandrillus sphinx*; 8) *Papio cynocephalus*; 9) *Saguinus niger*. Only 1 out of 12 female specimens revealed the presence of a *baubellum*, namely *Sapajus apella* (haplorrhine).

Therefore, total number of 3D models of *ossa genitalia* obtained (available at https://www.morphosource.org/Detail/ProjectDetail/Show/project_id/771) was 10.

**Fig 5:**
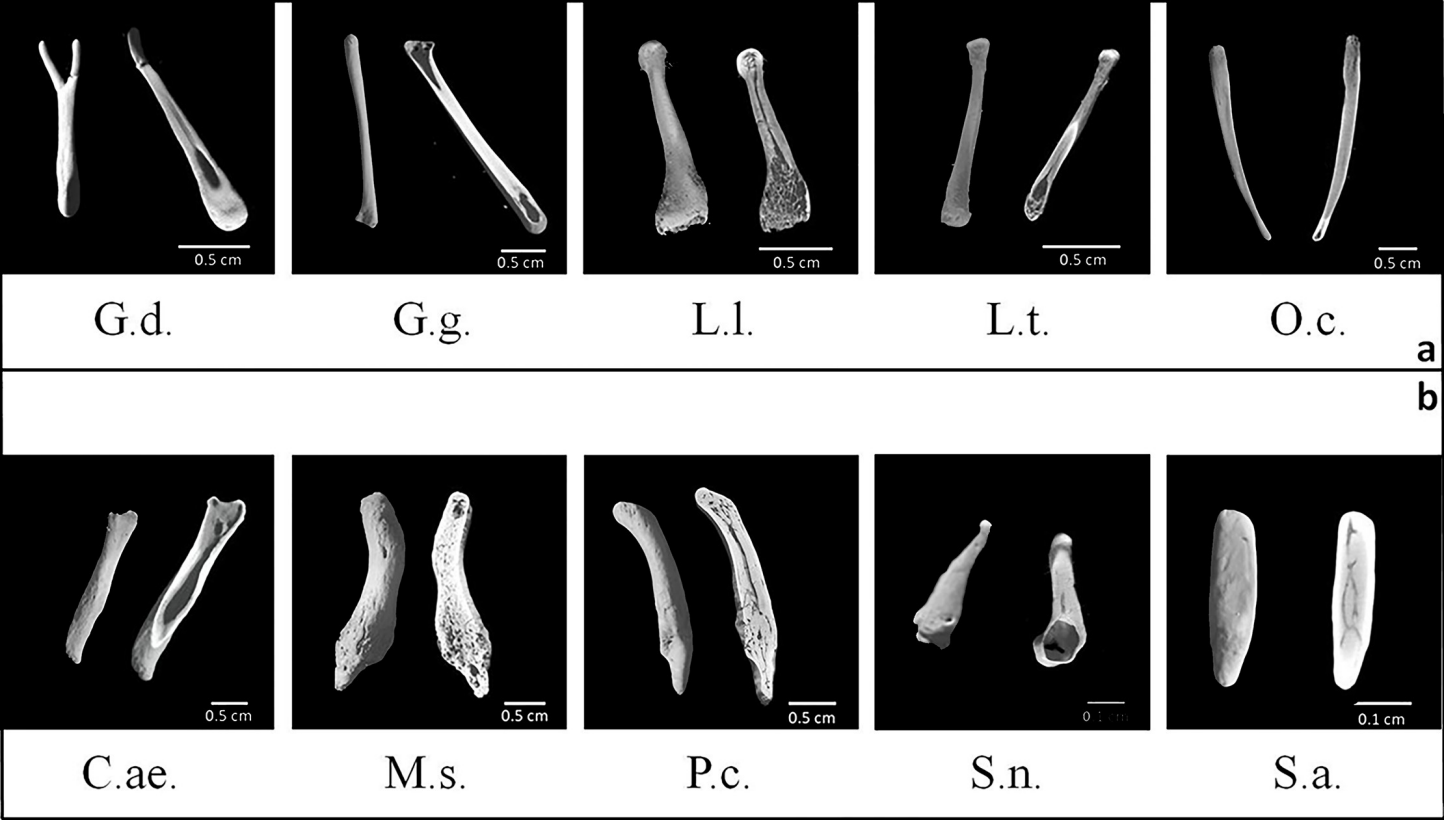
*Ossa genitalia* 3D external surface. 3D virtual volumes of the external surface (on the left) and internal structure (on the right) of 9 *bacula* (G.d., G.g., L.l., L.t., O.c., C.ae., M.s., P.c., S.n.) and 1 *baubellum* (S.a.). a = strepsirrhine species; b = haplorrhine species. Letters below each 3D image stay for specimens’ acronym (see Table 3).

**Fig 6:**
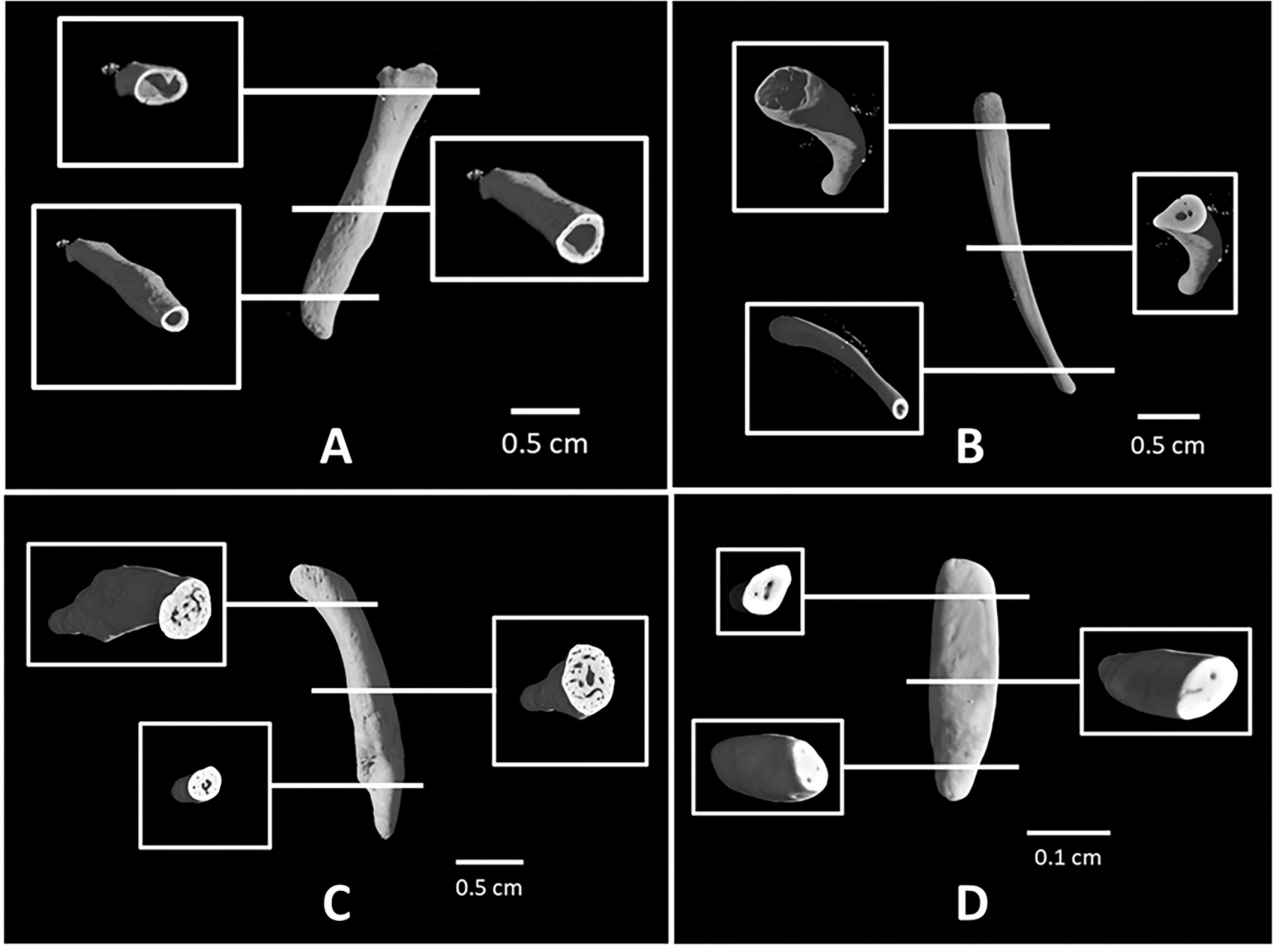
Types of primate *bacula*. 3D virtual volumes of 4 different types of *bacula*. For each type, internal structures and cross sections of epiphyses and diaphysis are shown. A: totally hollow structure (*Chlorocebus aethiops*). B: hollow epiphyses and solid diaphysis with few channels (*Otolemur crassicaudatus*). C: totally solid structure in both epiphyses and diaphysis with a network of channels and trabecular systems (*Papio cynocephalus*). D: totally solid structure of *baubellum* (*Sapajus apella*) with some channels.

Scanning was firstly devoted to genital bone reconstruction in order to investigate both surface and inner structure characteristics. Concerning the external surface, two different types were found, either rough with a jagged surface or very smooth and coherent; whereas for the bone internal structure, four main types were found (see Table 4 and Figs 5 and 6). A first type of bone had a totally hollow inner structure with neither trabecular-like systems nor labyrinthic system of channels inside (Figs 5 and 6A). By hollow we did not mean that bone is empty, rather that it does not contain anything radiopaque. However, only a destructive sampling could verify the presence (or not) of fatty bone marrow inside the bone. A second type would include two alternative versions: one having hollow epiphyses and solid diaphysis, with a few or several channels inside (Figs 5 and 6B), and another (*baculum* of *S*. *niger*) showing a hollow proximal epiphysis, but solid diaphysis and distal epiphysis. A third type was an entirely solid inner structure with intricate networks of trabecular-like systems and channels in both epiphyses and diaphysis (Figs 5 and 6C). Last type was a totally solid internal structure with only some channels (Figs 5 and 6D) which belong to a *baubellum* sample. Linear length data of each genital bone were reported in Table 4.

**Table 4:** Morphological characters of *ossa genitalia*.

Species	Acr.	Ext. Surf.	Int. Struct.	Position	L (mm)
***Galagoides demidoff***	**G.d.**	**smooth**	**hollow ep. solid diaph.**	**fill entire lenght**	**6.6**
(G. Fischer, 1806)					
***Galago gallarum***	**G.g.**	**smooth**	**hollow ep.solid diaph.**	**fill entire lenght**	**18.29**
Thomas, 1901					
***Loris lydekkerianus***	**L.l.**	**rough**	**hollow ep. solid diaph.**	**fill entire lenght**	**13.27**
Cabrera, 1908					
***Loris tardigradus***	**L.t.**	**rough**	**hollow ep. solid diaph.**	**fill entire lenght**	**9.16**
(Linnaeus, 1758)					
***Otolemur crassicaudatus***	**O.c.**	**smooth**	**hollow ep. solid diaph.**	**fill entire lenght**	**23.1**
(É. Geoffroy, 1812)					
***Chlorocebus aethiops***	**C.ae.**	**smooth**	**Hollow**	**distal half**	**13.82**
(Linnaeus, 1758)					
***Mandrillus sphinx***	**M.s.**	**rough**	**Solid**	**distal half**	**22.84**
(Linnaeus, 1758)					
***Papio cynocephalus***	**P.c.**	**rough**	**Solid**	**distal half**	**19.49**
(Linnaeus, 1766)					
***Saguinus niger***	**S.n.**	**rough**	**hollow ep. solid diaph.**	**distal half**	**2.99**
(É. Geoffroy, 1803)					
***Sapajus apella***	**S.a.**	**rough**	**solid**	**distal half**	**3.1**
Linnaeus, 1758					

For each detected bone total mean linear length (L), external surface (Ext. Surf.), internal structure (Int. Struct.; ep. = epiphyses; diap. = diaphysis) and bone position (Position) are described. For every species, acronym (Acr., same used for figures) is also reported.

**Fig 7:**
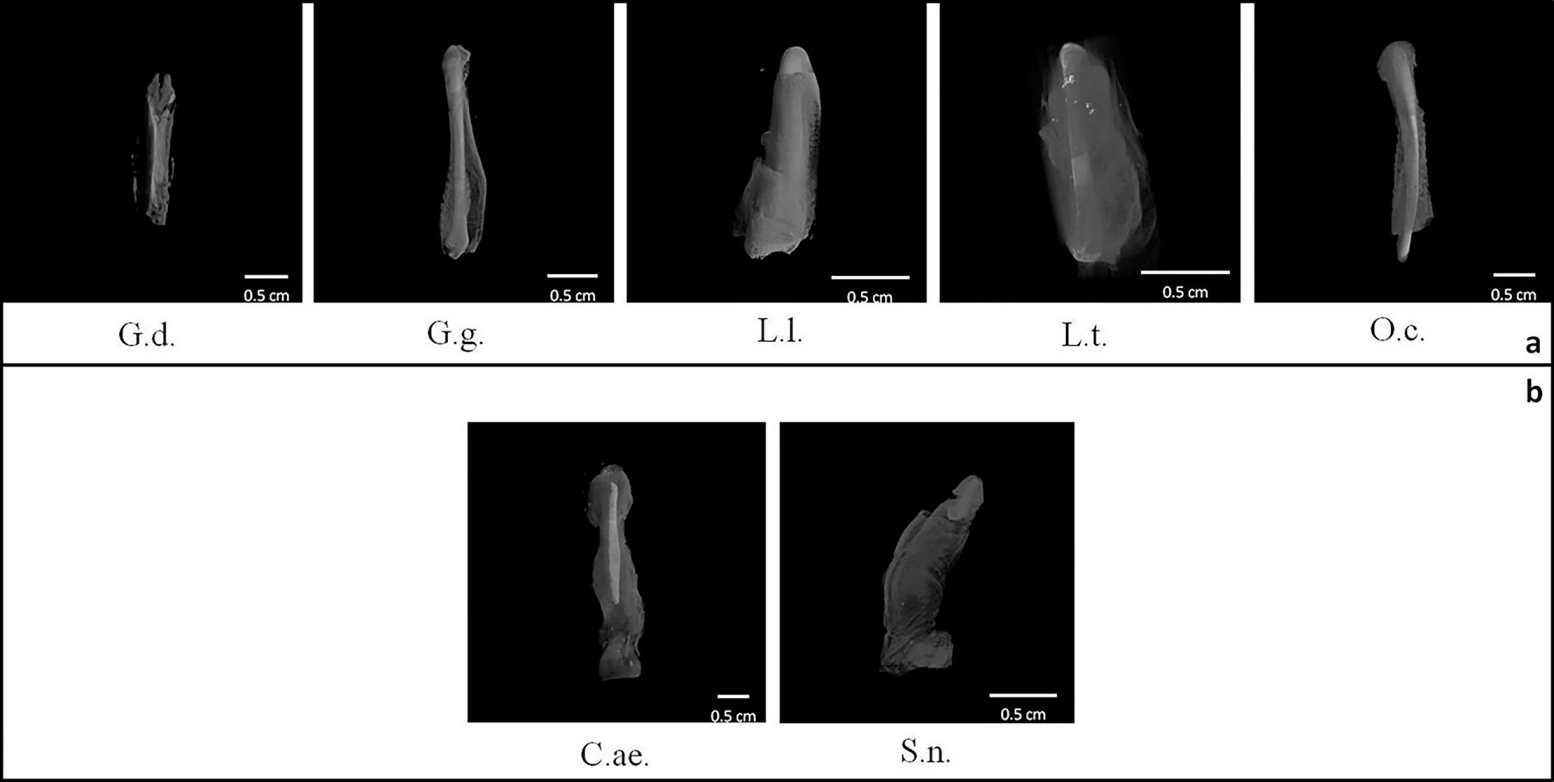
Positions of *bacula*. 3D view of external genitals with soft tissue transparency so to focus on *baculum* position (G.d., G.g., L.l., L.t., O.c., C.ae., S.n.). a = strepsirrhine species; b = haplorrhine species. Letters below each 3D image stay for specimens’ acronym (see Table 3).

**Fig 8:**
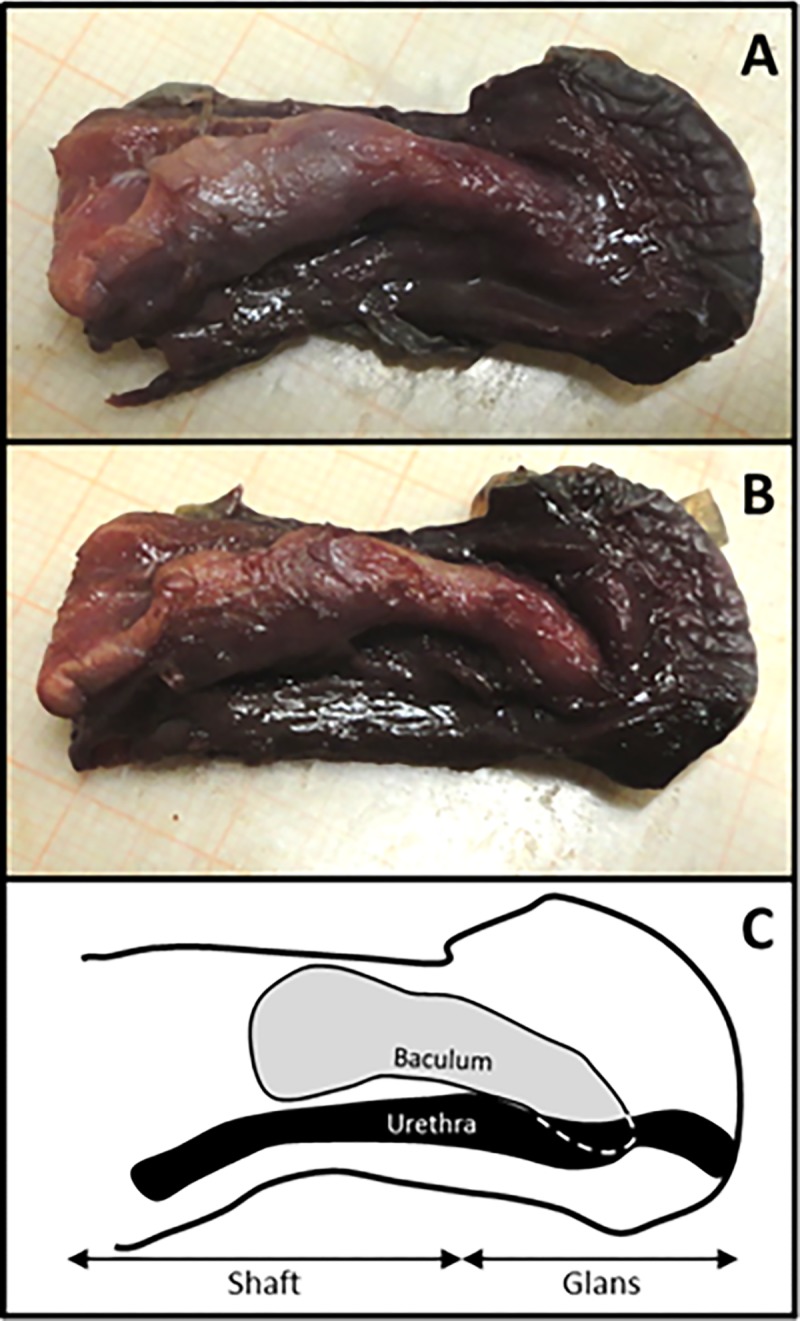
*Baculum* extraction. Dissection of *Mandrillus sphinx* penis for *baculum* extraction. A) sagittal section of penis, showing proximal epiphysis of *baculum*; B) secondary sagittal section, parallel to the first, showing both proximal and distal epiphyses of *baculum*; C) sketch of reciprocal anatomical relationships between *baculum* and urethra.

Secondly, scanned samples were also used to investigate the position of the bone inside the genitals. To do this, the area corresponding to peak of soft tissues which was disabled to let the bone appearing, was reduced and soft tissues appeared again in transparency (Table 4 and Fig 7). It was glaring the difference of *baculum* position between strepsirrhine and haplorrhine primates, the former showing *baculum* filling the entire length of penis, whereas in the latter genital bones were placed in the distal half of penis. Additional details about both *baculum* position and orientation inside the penis come from the manual bone extraction in one male *M*. *sphinx* in which a dorsal position relative to the urethra was revealed (longitudinal section of penis, Fig 8A). However, while the baculum proximal epiphysis (considering the *baculum* as a long bone, see below) was visible and dorsal to the urethra, the distal epiphysis could not be seen from that perspective. In fact, an apparent slight torsion of *baculum* along proximo-distal axis made the distal epiphysis, inside the glans (*i*.*e*., as Dixson suggested [44]), becoming lateral to the urethra, resulting in proximal and distal epiphyses on different sagittal planes (Fig 8B and 8C). A clear asymmetry was detected in *M*. *sphinx baculum* (S1 Fig).

### Technical notes

**Fig 9:**
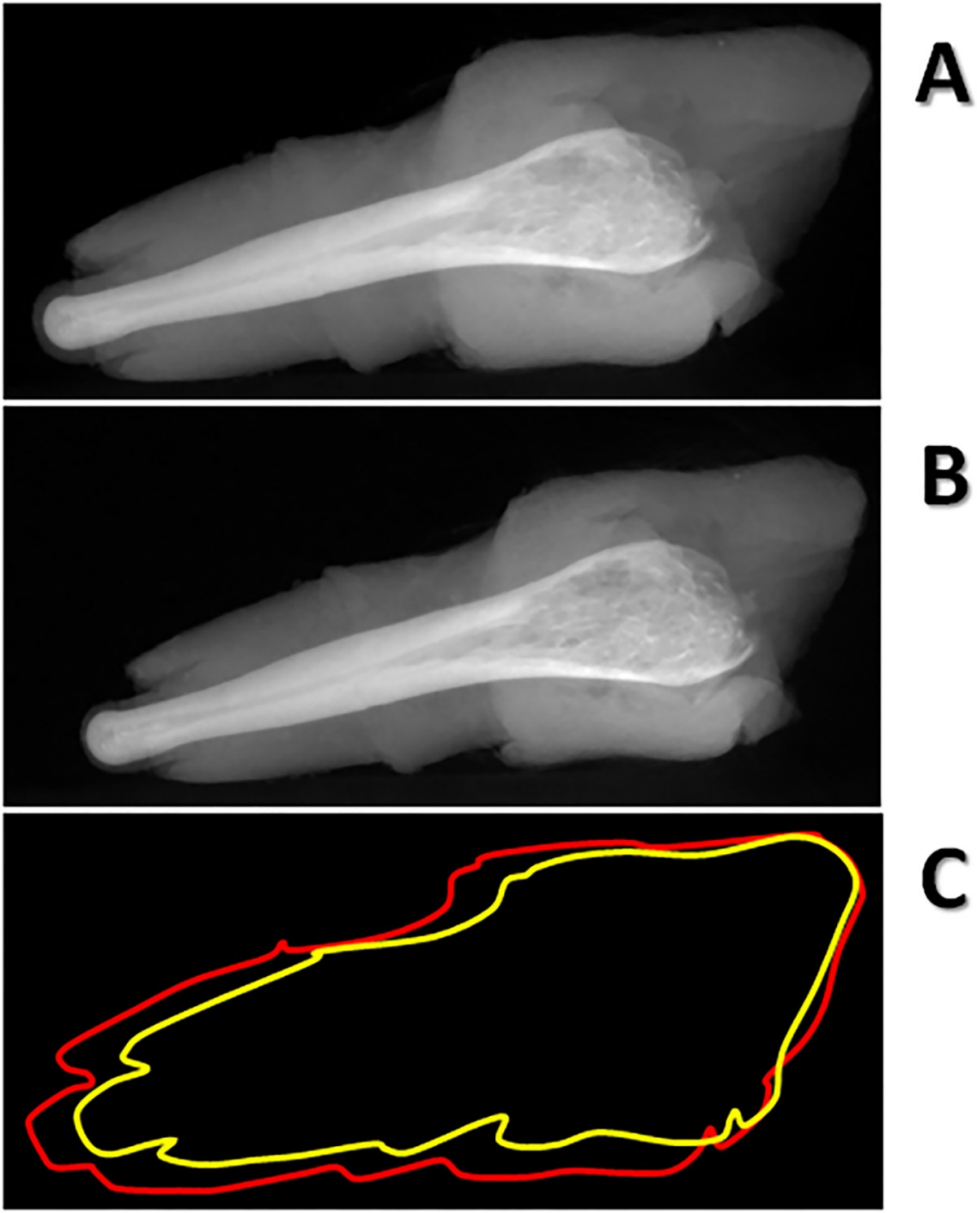
Sample volume reduction during the scan. Example of volume decrease occurred during the scan of a museum sample (*Loris lydekkerianus*) verified by comparing radiographic projection n° 0 (A) and n°899 (B) at the beginning and at the end of a tomographic acquisition process (900 radiographic projections acquired in an angular range of 360 degrees). The sample volume variation is shown (C) by overlaying outline of sample at the beginning of the scan (red line) and outline of sample at the end of the scan (yellow line).

- Although, unfortunately, *G*. *demidoff baculum* appeared broken (S2A Fig), it was possible to virtually repair it by using the imaging software (VG Studio Max 1.0 software) (S2B Fig) and consequently be able not to discard a precious sample.
- Comparing the first (Fig 9A) and the last (Fig 9B) radiographic projection of the genitals of *Loris lydekkerianus*, a mismatch appeared as if the sample was subjected to a (grisly!) repositioning. In fact, the overlaying of the red outline (radiographic projection of sample before the scan) and the yellow outline (radiographic projection of sample after the scan) revealed a sample volume reduction (Fig 9C, see discussion). The bidimensional blurry slices consequently obtained did not show suitable for 3D reconstruction.

## Discussion

While traditional approaches and methodologies would entail genital bone extraction from tissues and histology (i.e., a completely disruptive approach) in order to study their position, orientation, external surface and internal structure, innovative extant non-invasive techniques would not. In fact, depending on museum requirements (*e*.*g*., *in-situ* availability of micro-CT, museum willingness to loan samples), the micro-Computed Tomography turns out to be the most suitable and exhaustive modern technique to achieve the above mentioned results. In particular, micro-CT gives high resolution information about external surface and internal structure nevertheless making an unaltered sample available for future researchers.

Thanks to micro-CT, we recovered a high 3D-form variability: the “Y”-shaped *baculum* found in *G*. *demidoff*; the “stick”-*baculum* of *O*. *crassicaudatus*; the hook-*baculum* of *M*. *sphinx* and *P*. *cynocephalus* (both cercopithecids). Even more significantly, we disclosed for the first time the external and internal 3D morphology of 9 *bacula* for 9 species, and of a single *baubellum* as a tenth species. Differences detected in the internal structure, in particular, allowed to exclude the interpretation of *ossa genitalia* as a simple calcareous deposit and supported, instead, that of a living tissue having its own Haversian-like channels [13,52]. Four different internal structures clearly appeared, depending on presence/absence and concentration of trabecular- and Haversian-like systems: 1) a hollow *baculum*; 2) several *bacula* with solid epiphyses and hollow diaphysis; 3) two solid *bacula*; 4) a solid *baubellum*.

Although based on a small sample size, by comparing specimens to each other we detected two alternative positions of the *baculum* inside the penis: filling its entire length in Strepsirrhine species and placed in its distal half in Haplorrhine species. Despite no *baubella* were found in female strepsirrhines available, the single specimen of *os clitoris* scanned in the haplorrhine *S*. *apella* was placed inside the ending tip of clitoris, resembling haplorrhine *baculum* position. In previous anatomical studies, the positioning of both *baculum* and *baubellum* in the investigated species was either not reported, or explicitly stated in the text, or else simply inferable from drawings [24,31,35,41,52]. Position data, however, have never been emphasized as a discriminant trait between suborders and the only difference clearly reported is about length. In fact, it is possible that the longer *bacula* found in strepsirrhines [44] may contribute to the different position we observed relative to penis length. Our linear length measurements of scanned *bacula* supported that the longest one belongs to a strepsirrhine species (*Otolemur crassicaudatus*). In conclusion, in order to draw up a general pattern out of these individual observations and making it valid for the entire order of primates, differences between the positioning of the genital bones in Strepsirrhines and Haplorrhines must be confirmed by increasing sample size, for both *baculum* and *baubellum*. Asymmetry found in *M*. *sphinx baculum* should also find an appropriate context to be discussed and deepened.

The proposed 3-step protocol of investigation might be used firstly as a tool to obtain either simple (presence/absence) or complex (3D) data—depending on the study conditions and limitations—and secondly as a sample sorting procedure to select qualified specimens for micro-CT scanning which, as reported above, represent a time consuming procedure. Step 1: although proved useful in anesthetized living individuals (Carosi pers. obs.), manual palpation in museum specimens could either be hampered by the stiffness of dead tissues long preserved in either alcohol or formalin (e.g., female *S*. *niger* dating 1843 in our sample) or else fail to detect extremely small bones. Step 2: X-rays might represent a valuable tool if, exploring museum wet primate collections (i) a destructive sampling application is rejected, (ii) a micro-CT scanner is not available, and/or (iii) specimens available are only entire bodies too big to fit into the micro-CT scanner. Nevertheless, X-rays failed (one case) to discriminate between bone and radiopaque artefacts. Step 3: micro-CT allowed to (1) recognise a radiopaque artefact, (2) virtually repair any kind of bone fracture (recovering a precious otherwise useless sample), (3) investigate both external and internal structure of genital bones.

Nonetheless, in the case of fresh samples from primate remains (fated to be cremated), a traditional disruptive extraction protocol turned out to be useful to investigate more in detail position and orientation of bone, in particular as related to the urethra. This finding is particularly useful if considering that many old museum specimens, even if available for disruptive sampling, would unlikely be unsuitable for such investigation due to the formalin perfusion, *i*.*e*., the old traditional museum preservation technique. Primate cadavers should then be considered precious material as well as museum primate collections and should not be wasted. Ancient treatises on primate comparative anatomy [23,35,36,53] came from the sacrifice of many wild individuals which nevertheless allowed to carry out several studies about (but not limited to) morphology and anatomy. Nowadays all primate species are currently listed in the main international conventions for endangered fauna preservation, and collection of wild individuals for research purposes is not allowed anymore based on both ethical and conservation issues. However, national and international natural history museums still host many of those wild-caught individuals which still dramatically support investigations on anatomy, morphology, systematics and taxonomy. In this scenario, in order to preserve these precious collections, an increased application of non-invasive vs. invasive techniques, should be encouraged.

### Technical recommendations

During tomography scanning, it is mandatory that samples do not move in order to avoid resulting blurry images of slices not suitable for 3D reconstruction. In fact, for samples fixed in alcohol, evaporation may produce undesired specimen movements while scanning and, in addition, it may induce a reduction of tissues’ volume. In order to prevent all this to happen and based on empirical trials, we suggest two alternative preventive measures: 1) specimens may be removed from alcohol and sit overnight to let the alcohol evaporating before the micro CT‐scanning, and 2) before positioning the specimen on rotating turntable, it could be inserted in a small plastic bag which, briefly saturating with evaporated alcohol, will avoid the volume reduction.

We believe that advanced anatomical data about form, position and inner structure of genital bones in primates as those provided by micro-CT scanning technique might improve the study of the neglected topic of *ossa genitalia*. An investigation about relations between genital bone form variation and species reproductive and ecological correlates, in a phylogenetic perspective, might help unravelling further functional and evolutionary hypotheses. In addition, virtual data about genital bone surface could be analyzed by using either geometric morphometric techniques (for example following the same landmark and semi-landmark method applied to investigate molluscan shell shape variation by [54–56]) or other methods, such as the finite element analysis [57–61] and “alpha-shapes” [62]. In conclusion, micro-CT technique proved appropriate to discover knowledge still hidden in museum specimens or fresh cadavers, preventing them not only to be stored in a closet or else get trashed after necropsy, rather allowing them to still contribute to science and live a second life.

## Supporting information

S1 Table List of specimens collected for this study. For every specimen, scientific name, taxonomic suborder (S = Strepsirrhini; H = Haplorrhini), sex (M = male; F = female), provider (NHMLS = Natural History Museum "La Specola"; IZSLT = Istituto Zooprofilattico Sperimentale di Lazio e Toscana; CMZ = Civic Museum of Zoology), catalogue code (only available for museum specimens, CC), city of provider and age class (also in months, if available) are reported.
(XLSX)

S1 Fig Asymmetry in a primate *baculum*. Example of *baculum* asymmetry in *Mandrillus sphinx* (ventral view). R = right; L = left; D = distal; P = proximal.
(TIF)

S2 Fig Virtual reassembly of a broken baculum. 3D volume of *Galagoides demidoff* baculum: A) bone fracture in the distal half of sample; B) the same bone virtually repaired.
(TIF)
