# Supplementary figures and images for: A 3D journey on virtual surfaces and inner structure of ossa genitalia in Primates by means of a non-invasive imaging tool
Source: PLoS One. 2020 Jan 30;15(1):e0228131. doi: 10.1371/journal.pone.0228131 (PMC6992188; doi:10.1371/journal.pone.0228131)

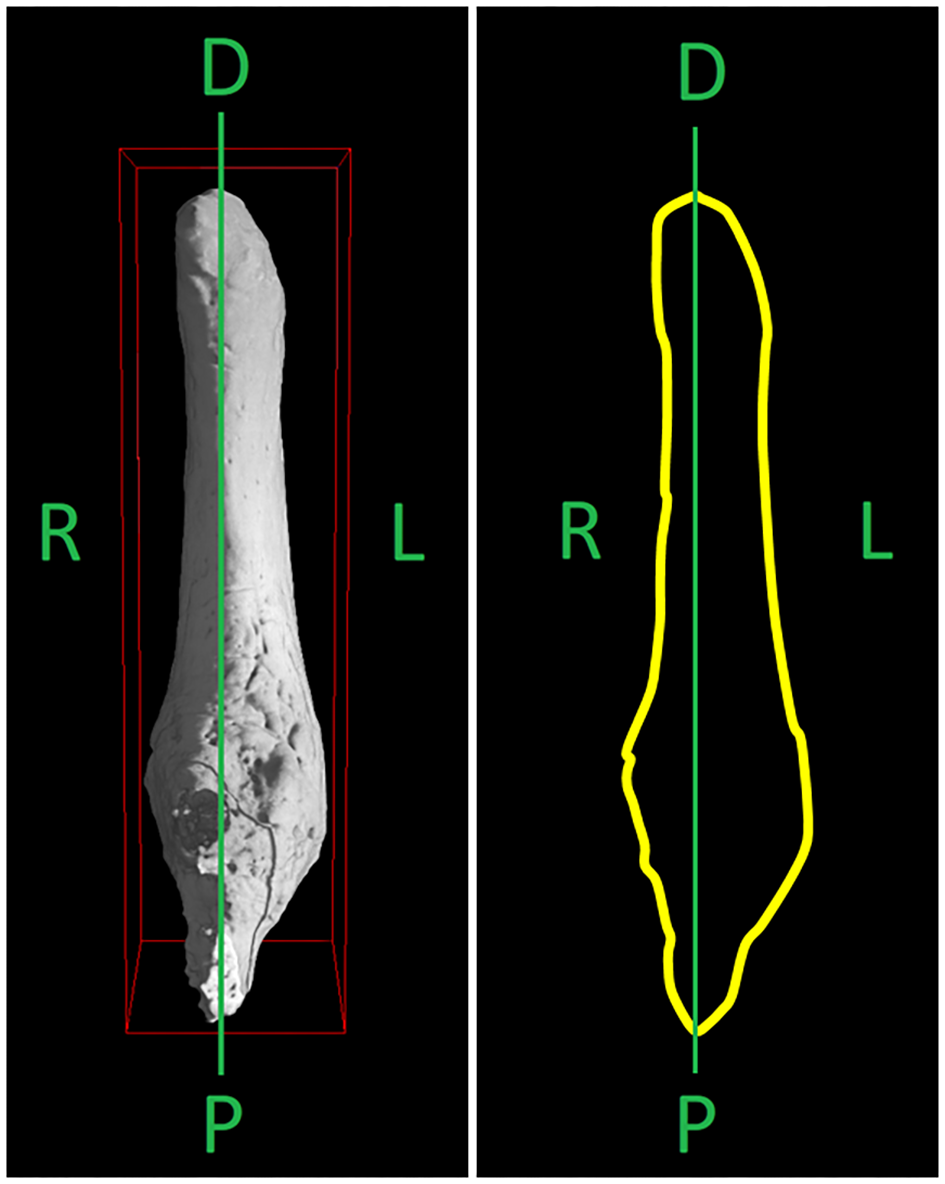

Supplement: S1 Fig — Example of baculum asymmetry in Mandrillus sphinx (ventral view). R = right; L = left; D = distal; P = proximal. (TIF) [file pone.0228131.s002.tif]

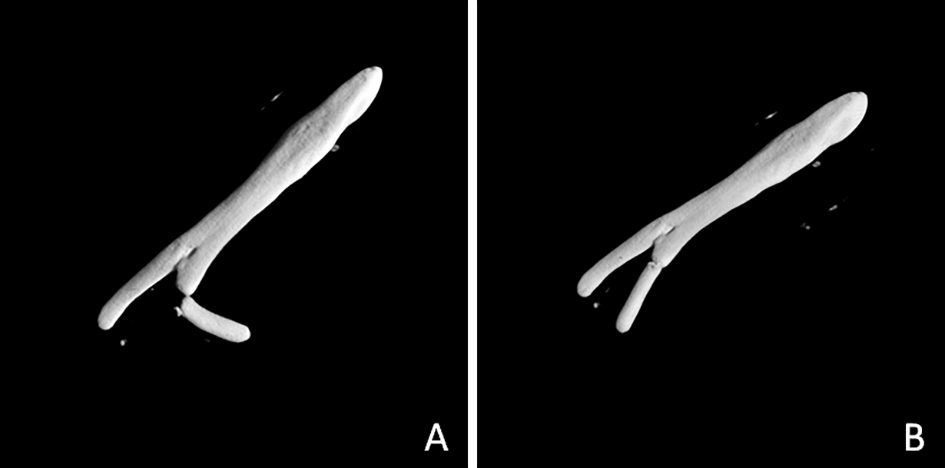

Supplement: S2 Fig — 3D volume of Galagoides demidoff baculum: A) bone fracture in the distal half of sample; B) the same bone virtually repaired. (TIF) [file pone.0228131.s003.tif]
